# Direct and Dynamic Detection of HIV-1 in Living Cells

**DOI:** 10.1371/journal.pone.0050026

**Published:** 2012-11-28

**Authors:** Jonas Helma, Katrin Schmidthals, Vanda Lux, Stefan Nüske, Armin M. Scholz, Hans-Georg Kräusslich, Ulrich Rothbauer, Heinrich Leonhardt

**Affiliations:** 1 Department of Biology II, Ludwig Maximilians University Munich, Planegg-Martinsried, Germany; 2 Department of Infectious Diseases, Virology, University Hospital Heidelberg, Heidelberg, Germany; 3 Livestock Center of the Faculty of Veterinary Medicine, Ludwig Maximilians University Munich, Oberschleissheim, Germany; 4 Center for Integrated Protein Science, Munich, Germany; 5 ChromoTek GmbH, Planegg-Martinsried, Germany; International Centre for Genetic Engineering and Biotechnology, Italy

## Abstract

In basic and applied HIV research, reliable detection of viral components is crucial to monitor progression of infection. While it is routine to detect structural viral proteins *in vitro* for diagnostic purposes, it previously remained impossible to directly and dynamically visualize HIV in living cells without genetic modification of the virus. Here, we describe a novel fluorescent biosensor to dynamically trace HIV-1 morphogenesis in living cells. We generated a camelid single domain antibody that specifically binds the HIV-1 capsid protein (CA) at subnanomolar affinity and fused it to fluorescent proteins. The resulting fluorescent chromobody specifically recognizes the CA-harbouring HIV-1 Gag precursor protein in living cells and is applicable in various advanced light microscopy systems. Confocal live cell microscopy and super-resolution microscopy allowed detection and dynamic tracing of individual virion assemblies at the plasma membrane. The analysis of subcellular binding kinetics showed cytoplasmic antigen recognition and incorporation into virion assembly sites. Finally, we demonstrate the use of this new reporter in automated image analysis, providing a robust tool for cell-based HIV research.

## Introduction

Over the last decades, a large number of HIV (Human Immunodeficiency Virus) detection methodologies have been developed. Such techniques include *in vitro* based approaches to measure primary infection in patients, for example by detecting HIV-specific antibodies or by directly detecting HIV-derived, structural proteins (e.g. the capsid protein CA/p24). Cell-based HIV detection relies on molecular imaging techniques, such as immunofluorescence, and electron microscopy, which both allow direct visualization of viral structures but require cell fixation. Live cell reporter systems include the implementation of genetic reporter elements that get activated upon HIV infection [1], [2], [3] as well as recombinant viruses, where tags or fluorescent proteins have been integrated to study replication dynamics in living cells. In particular, HIV assembly processes in living cells have been a major subject of investigation over the last years (recently reviewed [4], [5]). HIV-1 virion assembly is orchestrated by the viral polyprotein Gag. Gag consists of an N-terminal matrix domain (MA) that mediates membrane attachment, an internal capsid domain (CA) that mediates multimerization of Gag, a nucleocapsid domain (NC) that binds and packages the viral RNA genome and a C-terminal p6 peptide that is involved in virus budding and release. Upon virion budding, Gag gets proteolytically processed by the viral protease and subdomains are released as functional proteins within mature virions. In principle, genetically encoded tags for live cell imaging purposes may be integrated at various sites within the Gag polyprotein. For example, C-terminal insertion of the green fluorescent protein (GFP) as well as internal insertion at the C-terminus of the MA domain allows dynamic visualization of the assembly of virus like particles (VLPs) [6], [7], [8]. The latter insertion site proved particularly compatible with viral replication and has been used for different tagging strategies, including biarsenical-tetracysteine tagging and SNAP-tagging [9], [10].

Having such tools at hands, various modern light microscopy techniques, including widefield, confocal and total internal reflection fluorescence microscopy, have been used to investigate the HIV assembly process at both single-cell and single virion level, elucidating the spatiotemporal dynamics of HIV morphogenesis and demonstrating molecular interactions with viral and host factors [11], [12], [13], [14]. Moreover, novel live-cell super-resolution imaging techniques [15], [16] will likely open new possibilities to study fluorescently labeled viruses. However, all these new imaging techniques rely on recombinant viral fusion proteins while the direct visualization of genetically unmodified HIV still remained elusive.

The recent development of fluorescent intracellular single domain nanobodies, so-called chromobodies [17], [18], [19], offers a general approach for dynamic detection and visualization of virtually any natural and genetically unmodified factor in living cells.

Here, we describe a high affinity chromobody that allows direct and dynamic visualization of HIV-1 formation in living cells.

## Results

### Generation of a CA-specific nanobody

**Figure 1:**
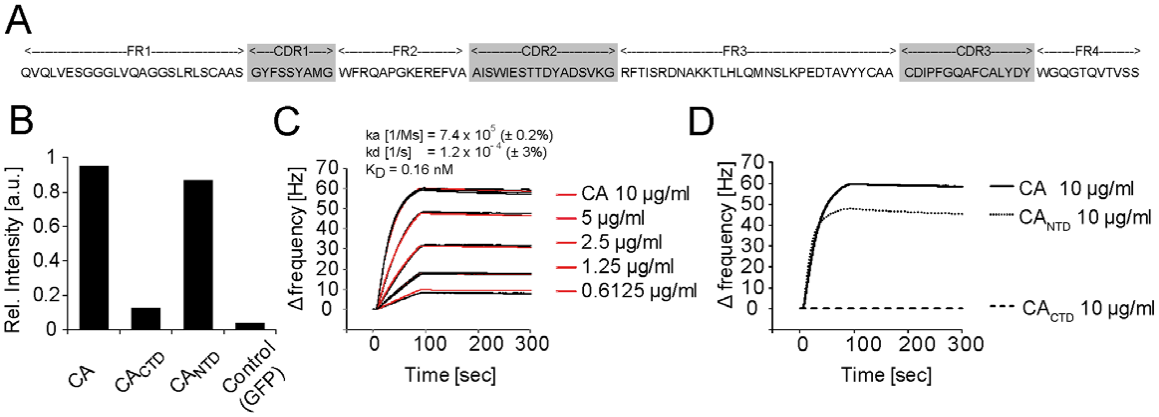
Identification and *in vitro* characterization of a CA-specific nanobody. (**A**) Amino acid sequence of CA_NTD_cb1. (**B**) CA_NTD_cb1 was tested for antigen specificity by solid phase phage ELISA, showing significant binding response for full-length CA and CA_NTD_ but no binding to CA_CTD_. Purified GFP was used as negative control. Bars show representative values of technical duplicates. (**C**) Affinity measurements with a quartz crystal microbalance (QCM) system. Data fitting (red lines) and rate constant calculation was carried out with ClampXP analysis software. (**D**) To further measure and confirm binding specificity, purified full-length CA, CA_NTD_ or CA_CTD_ were injected at 10 µg/ml and passed over immobilized CA_NTD_nb1.

In a first step to generate a nanobody reporter for HIV-1 detection in living cells, an alpaca was immunized with purified HIV-1 CA protein and a phagemid library was generated, representing the respective VHH (nanobody) repertoire. Three subsequent phage display cycles revealed an enrichment of one VHH sequence (**Figure 1a**). Antigen recognition and subdomain specificity was tested in a solid phase phage-ELISA with purified CA, the isolated N-terminal domain of CA (CA_NTD_) and the isolated C-terminal domain of CA (CA_CTD_), indicating specific CA binding and a binding preference for CA_NTD_ (**Figure 1b**). For further binding analysis *in vitro*, the nanobody, termed CA_NTD_cb1, was cloned with a C-terminal 6×His-tag, expressed in *E. coli* and purified with immobilized metal ion affinity chromatography and size-exclusion chromatography. To determine the binding affinity, purified CA_NTD_cb1 was tested in a continuous-flow Quartz Crystal Microbalance (QCM) system. Affinity measurements with full-length CA resulted in a K_D_ value of 0,16 nM (**Figure 1c**), which is comparable to binding affinities of conventional antibodies [20]. Further binding measurements with isolated CA domains confirmed specific binding to CA_NTD_ (**Figure 1d**).

### CA_NTD_cb1 colocalizes with HIV structures at the plasma membrane

**Figure 2:**
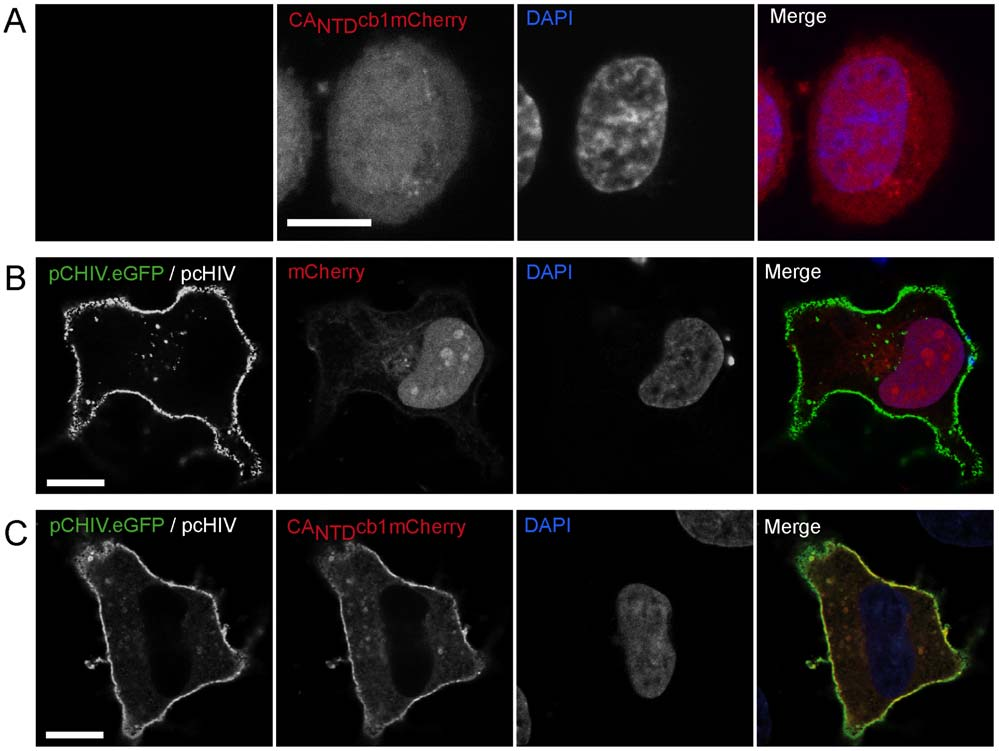
Intracellular antigen recognition. (**A**) mCherry-labelled CA_NTD_cb1, expressed in HeLa-Kyoto cells, is diffusely distributed throughout the cell. (**B**) GFP-labelled HIV-1 Gag is focally enriched at the plasma membrane. mCherry was used as control. (**C**) Upon co-expression, mCherry labelled CA_NTD_cb1 colocalizes with GFP-labelled HIV-1 Gag structures enriched at the plasma membrane. Scale bars, 10 µm.

Next, we set out to develop live cell detection of HIV-1 and in particular of the CA domain in the HIV-1 Gag polyprotein. For this purpose, we genetically fused the coding region of the CA-specific nanobody to fluorescent proteins, generating so-called chromobodies [17]. Expression of these constructs in HeLa-Kyoto cells showed ubiquitous, predominantly diffuse distribution, indicating that they do not interact with cellular structures (**Figure 2a**). To test intracellular binding of CA_NTD_cb1 to viral structures, HeLa-Kyoto cells were transfected with plasmids encoding all HIV-1 proteins (except Nef), giving rise to HIV-1-like particles with eGFP embedded in the MA domain of Gag [7], [8]. Cells were then co-transfected with constructs, carrying either mCherry (**Figure 2b**) or CA_NTD_cb1 coupled to mCherry (**Figure 2c**). mCherry co-transfected cells showed discrete focal structures of GFP-labelled Gag primarily at the plasma membrane, likely corresponding to assembly sites with mCherry signal being distributed throughout the cell. Importantly, CA_NTD_cb1 co-localized with viral assembly structures at the plasma membrane, indicating specific binding, but no significant interference with Gag membrane targeting in living cells.

### CA_NTD_cb1 directly detects individual HIV-1 assembly sites

**Figure 3:**
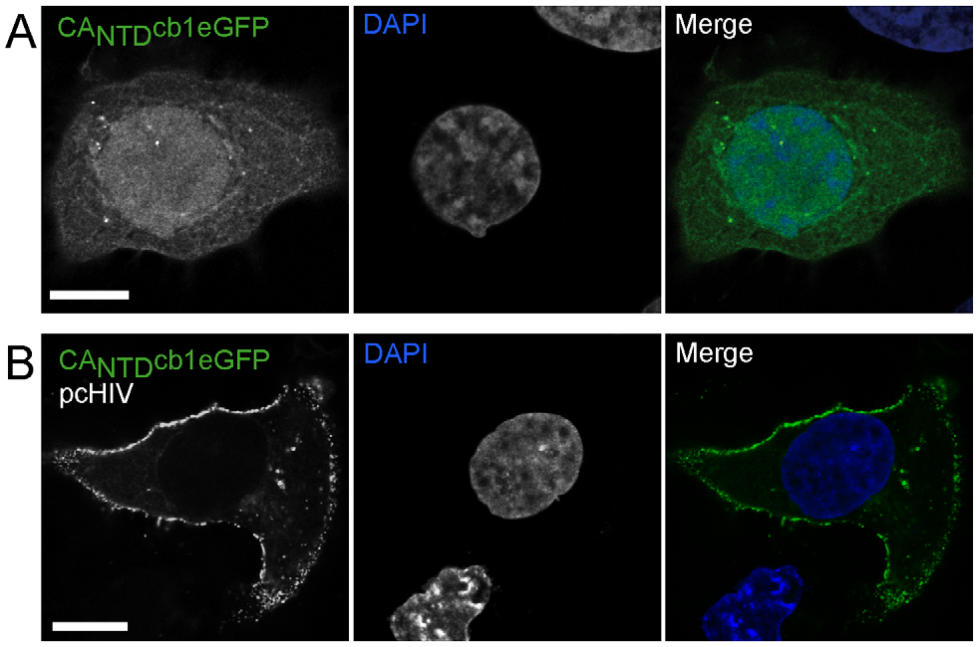
Detection of untagged HIV-1 Gag. (**A**) Subcellular distribution of eGFP-labelled CA_NTD_cb1, expressed in HeLa-Kyoto cells. (**B**) Upon co-expression with untagged HIV-1 Gag, eGFP-labelled CA_NTD_cb1 specifically localizes at focal structures enriched at the plasma membrane. Scale bars, 10 µm.

Since CA_NTD_cb1 colocalized with GFP-tagged viral structures, we anticipated it to be well suited for visualizing even untagged antigen in living cells. Thus, HeLa-Kyoto cells were co-transfected with a non-infectious plasmid pcHIV, producing non-labelled HIV particles, together with a plasmid encoding CA_NTD_cb1 coupled to eGFP, and were then subjected to confocal light microscopy. Co-expression resulted in specific localization of CA_NTD_cb1 to discrete focal structures at the plasma membrane (**Figure 3b**), similar to the GFP-labelled HIV structures, strongly indicating that CA_NTD_cb1 allows direct visualization of HIV-1 Gag assemblies, Co-expression of CA_NTD_cb1 did not abolish particle release, indicating that its binding to Gag is compatible with virion morphogenesis, while effects on infectivity cannot be excluded.

**Figure 4:**
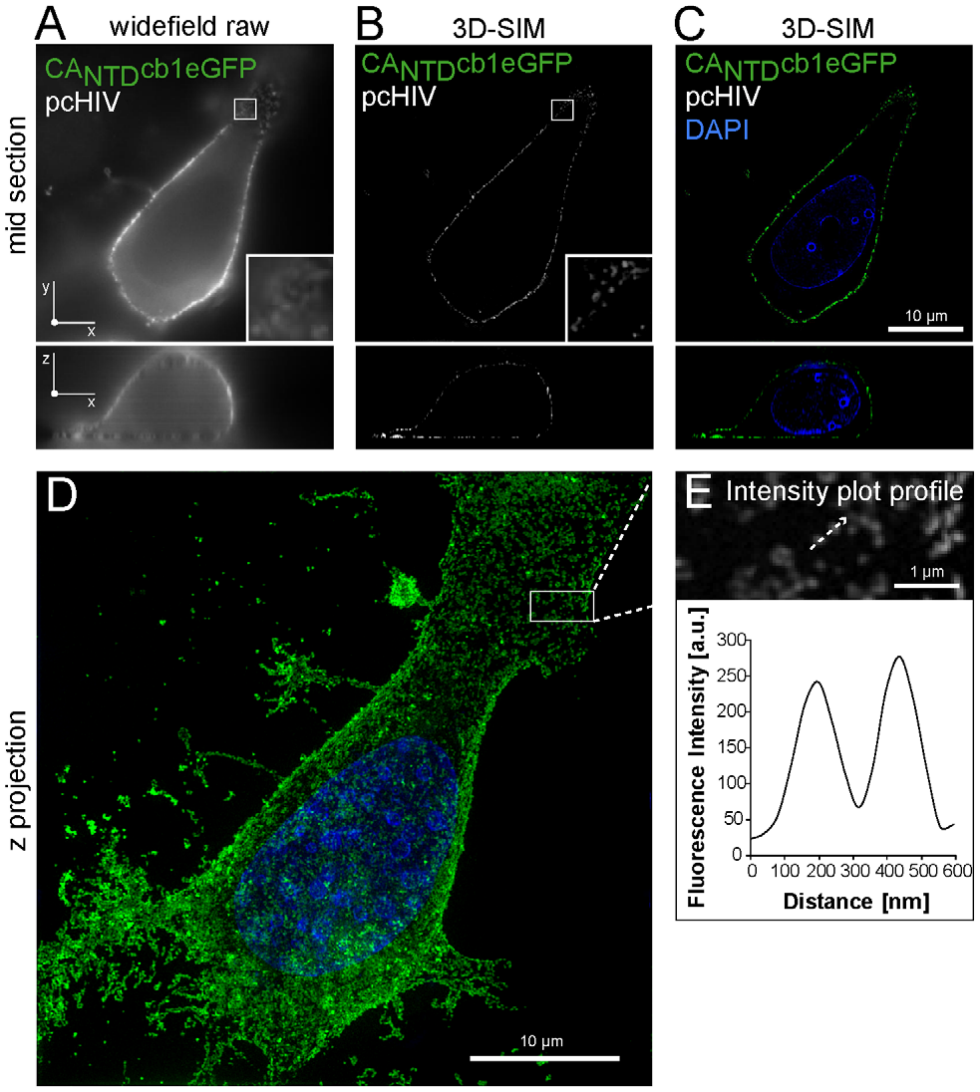
Super-resolution microscopy with 3D-SIM. (**A, B, C**) Widefield vs. 3D-SIM imaging. (**A**) For comparison, the raw widefield image of a HeLa-Kyoto cell, expressing CA_NTD_cb1eGFP and unlabelled HIV-1 Gag is shown. Zoom regions show higher-detail illustration of a membranous region. The bottom image shows a mid cross section in z and x dimension. (**B, C**) The same cell shown in (A), imaged using structured illumination. Shown are confocal mid sections displayed in grayscale (B, eGFP) or false colors (C, eGFP, DAPI). Scale bar, 10 µm. (**D**) Shown are 3D-rendered images of the cell shown in b–c, illustrating ubiquitous distribution of viral foci at the plasma membrane. (**E**). Intensity plot profiles over two representative CA_NTD_cb1-stained foci show distinct peaks in a 130–160 nanometer size range. Scale bar, 1 µm.

Detailed analyses of these focal structures by conventional light microscopy techniques are limited by diffraction and mediate resolution of subcellular structures to max. 200 nm. As a consequence, individual viruses with a size of about 100–150 nm, such as HIV-1, cannot be resolved. The recent development of super-resolution microscopy systems, including 3D-Structured Illumination Microscopy (3D-SIM) [21], [22], overcomes this analytical limitation. Thus, to further validate the nature of HIV-1 structures, directly stained with CA_NTD_cb1 in living cells, we performed 3D-SIM on preserved formaldehyde-fixed HeLa-Kyoto cells. For reasons of comparison, a conventional widefield image was recorded, exhibiting signal enrichment at the cellular periphery with strong out-of-focus blur (**Figure 4 a**). In the 3D-SIM image, showing a cellular mid section, the membranous structures are resolved as individual punctae with low background signal, suggesting the detection of individual viral assembly sites (**Figure 4 b, c**). The projection of 120 cross sections demonstrates the ubiquitous distribution of viral assemblies over the entire cell surface (**Figure 4 d**). Intensity plot profiling over representative punctae revealed a size range of about 130–160 nm for these structures, strongly indicating that CA_NTD_cb1 is detecting discrete, individual HIV assembly sites at the plasma membrane (**Figure 4 e**).

### Live cell analysis of HIV assembly

**Figure 5:**
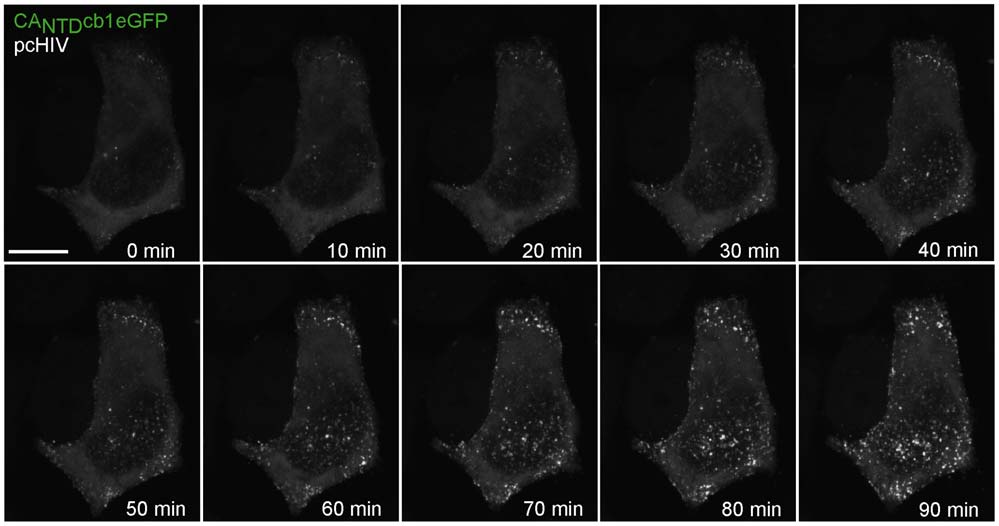
Visualizing the formation of HIV-1. A HeLa-Kyoto cell, expressing CA_NTD_cb1eGFP and HIV-1 Gag was monitored for 90 min at 1 min time intervals using confocal spinning disk microscopy (see also Movie S1). Shown are projections of 12 z-sections per time point. Scale bar, 10 µm.

**Figure 6:**
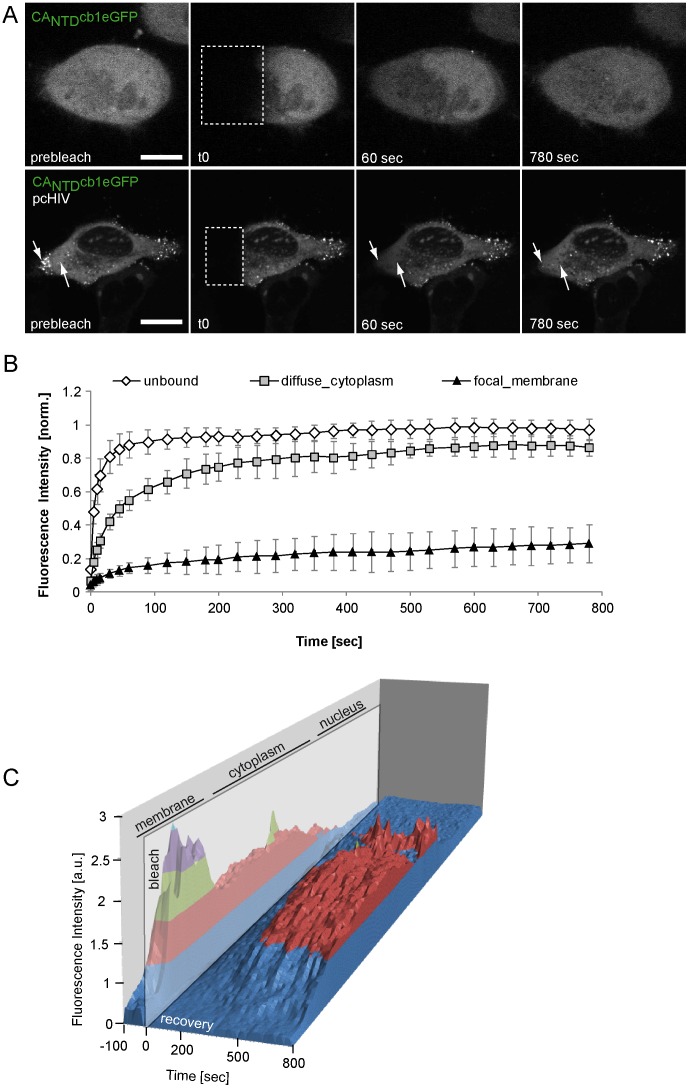
FRAP analysis of subcellular chromobody populations. (**A**) Fluorescence recovery of CA_NTD_cb1eGFP over time, either expressed alone (upper panel) or in combination with HIV-1 Gag (lower panel). Arrows in the lower panel indicate two regions of interest: Diffusely distributed CA_NTD_cb1eGFP signal in the cytoplasm and focal structures at the outer cellular rim, indicating virion assembly sites at the membrane. Scale bar, 10 µm. (**B**) Quantitative FRAP analysis reveals different mobility states of unbound and ubiquitous, diffusely and cytoplasmic as well as focal and membranous CA_NTD_cb1eGFP signal. Errors are standard 0deviations (n≥7). (**C**) 3D-FRAP representation of the same cell shown in (A) (lower panel), illustrating cytoplasmic recovery to almost initial level, while cumulated, membranous signal shows no recovery.

HIV-1 Gag assembly in living cells is described as a rapid transition from a soluble, diffusely distributed form in the cytoplasm to a focal and multimeric form that predominantly localizes at the plasma membrane [12], [13]. To test, whether it is possible to resolve this dynamic event using CA_NTD_cb1 for detection, HeLa-Kyoto cells were co-transfected with pcHIV and CA_NTD_cb1eGFP and subjected to live cell confocal spinning disk microscopy. Indeed, the overall distribution of CA_NTD_cb1eGFP signal rapidly changed from diffuse to focal, indicating HIV assembly (**Figure 5**). Figure 5 shows the projections of 12 z-sections recorded at each time point. Single confocal sections of this time series clearly indicate that assembly processes occur predominantly at the plasma membrane (Movie S1), consistent with previous reports [12], [23]. The shift to a predominant cytoplasmic distribution of CA_NTD_cb1 at the first time point in Figure 3 indicated binding to HIV-1 Gag. To measure the subcellular mobility of CA_NTD_cb1 and discriminate between different antigen-dependent organizational states, we performed Fluorescence Recovery After Photobleaching (FRAP) experiments. As expected, CA_NTD_cb1 alone recovered rapidly (**Figure 6a, b**). Upon co-expression with HIV-1 Gag, cytoplasmic, diffusely distributed CA_NTD_cb1 was still mobile, but recovered significantly more slowly than the chromobody alone, indicating specific antigen recognition in the cytoplasm (**Figure 6a, b, c**). In contrast, FRAP analysis of focal structures at the plasma membrane showed almost no fluorescence recovery, suggesting specific and practically irreversible incorporation of CA_NTD_cb1 into viral assemblies (**Figure 6a, b, c**).

### CA_NTD_cb1 allows automated realtime high content analysis of HIV-1 morphogenesis

**Figure 7:**
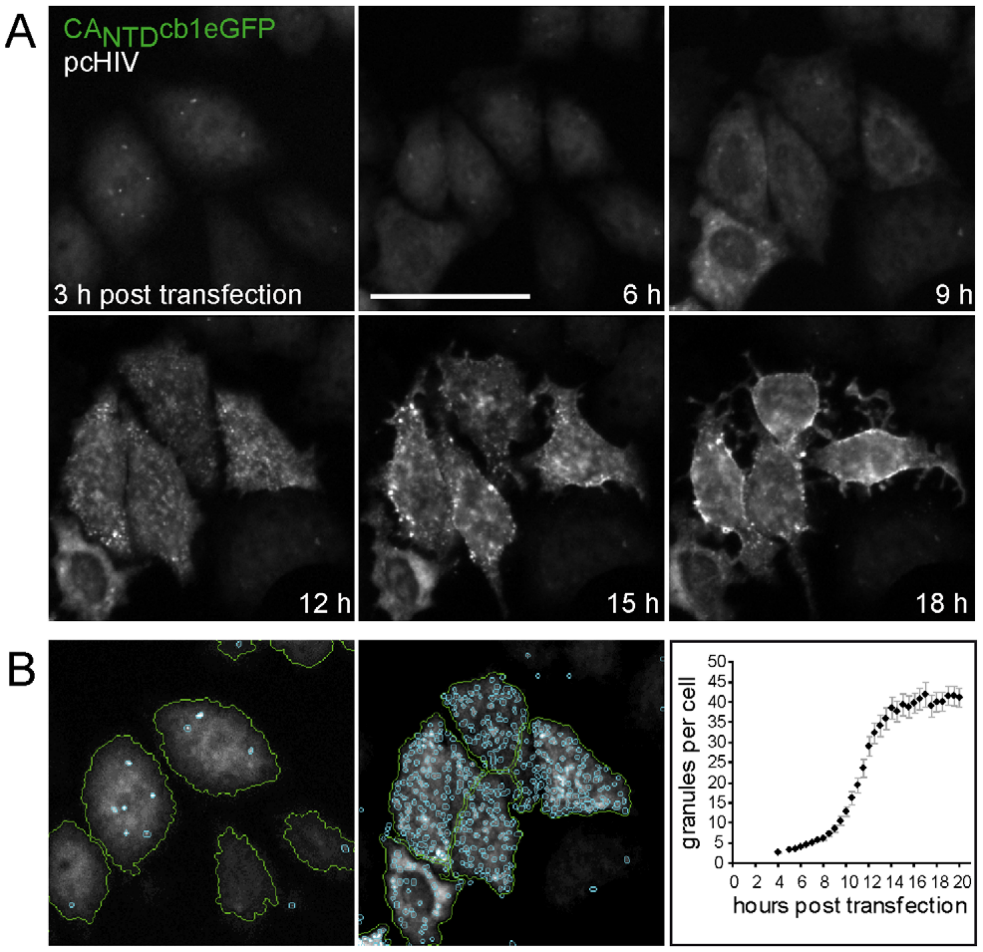
Automated monitoring of HIV morphogenesis. (**A**) HeLa-Kyoto cells, stably expressing CA_NTD_cb1eGFP were transfected with pcHIV, encoding untagged Gag and monitored by time lapse epifluorescence microscopy for 18 h at 10 min time intervals. Scale bar, 50 µm. (**B**) Automated pattern recognition for High Content Analysis. eGFP fluorescence was used to determine borders of individual cells by intensity threshold segmentation, indicated by green lines. Particles within cells were segmented and defined by size and intensity threshold setting, indicated by cyan coloured lines. Scale bars, 50 µm. The average number of granules per cell was determined and plotted over time. Error bars represent the standard error of the mean (n∼400 cells).

Modern cell-based imaging techniques aim at automated image analysis to extract a maximum of information from cellular assays. However, such High-Content Analysis (HCA) crucially relies on robust detection reagents. To test the application of CA_NTD_cb1 in HCA, we generated a HeLa-Kyoto cell line, stably expressing CA_NTD_cb1eGFP. These cells were then transfected with pcHIV and subjected to automated time-lapse imaging for a period of 18 hours after transfection. About 6–9 hours after transfection, chromobody distribution changed from ubiquitous to cytoplasmic localization. Subsequently, the distribution pattern changed from diffuse to focal structures accumulating at the membrane (**Figure 7a**). We found that this diffuse versus focal pattern is well suited for quantitative automated image analysis allowing the objective quantification of assembly processes in large cell numbers. For this purpose, the cytoplasmic fluorescence was used to define cellular boundaries within which granules were identified by intensity threshold segmentation and quantitatively monitored over time (**Figure 7b**), demonstrating the usefulness of nanobody-based, dynamic HIV detection in high content imaging.

## Discussion

Here, we describe a new biosensor that allows detection and dynamic tracing of HIV in living cells. This chromobody (CA_NTD_cb1) specifically recognizes the HIV-1 CA protein domain and detects individual assembly sites as shown with super-resolution microscopy. Live cell analysis demonstrated direct and dynamic visualization of HIV assembly processes by CA_NTD_cb1 and FRAP kinetic analysis revealed different mobility states, likely corresponding to different organizational states of HIV-1 Gag molecules during virion morphogenesis.

The development of Highly Active Antiretroviral Therapies (HAART), targeting the HIV-1 replication cycle at various stages has successfully constrained the pandemic AIDS progression during the last years and decades. Nonetheless, it is known that HIV-1 strains acquire multi-drug resistances, creating a constant need for novel inhibitory compounds. Virion assembly processes, visualized by CA_NTD_cb1, appear well suited for automated image analysis in HCA assay systems and thus provide a read-out for targeted antiviral compound screens. Furthermore, the chromobody-based detection described here should be a versatile tool to directly compare different HIV-1 variants, including primary isolates and viruses carrying mutations in functional sites.

In HIV cell biology, recent analyses of the dynamics of HIV-1 biogenesis have provided new insights into the regulation of HIV-1 Gag trafficking, interaction with cellular co-factors and formation of single virions [4], [12], [13], [14]. Structural analyses have revealed the intermolecular connectivity interfaces that coordinate the assembly process of the mature HIV capsid at atomic resolution [24], [25], [26], [27], [28], [29]. Very recently, a cryo-electron microscopy study revealed the molecular organization of the retroviral capsid in the immature state at subnanometer resolution [30], demonstrating remarkable, structural flexibility within the capsid. However, little is known about the dynamic contributions of the N- and C-terminal CA subdomains during assembly. We found that CA_NTD_cb1 specifically binds to the N-terminal domain of the HIV-1 CA protein, which is involved in Gag-Gag interactions during virion assembly. We did not observe any obvious defects but cannot entirely exclude interference with virion morphogenesis caused by the CA_NTD_cb1 chromobody. The dynamics, structural flexibility and robustness of capsid assembly are important topics of HIV biology. Generation of additional chromobodies with different binding sites should enable kinetic and functional studies to specifically probe the HIV assembly process.

## Materials and Methods

### VHH Libraries and Screening

Alpaca immunizations with purified HIV-1 CA protein, VHH-library construction and selection of CA-binding nanobodies were carried out as described previously [17] and have been approved by the government of Upper Bavaria (Permit number: 55.2-1-54-2531.6-9-06). In brief, 6 weeks after immunization, ∼100 ml blood were collected and lymphocytes were isolated by Ficoll gradient centrifugation. Total RNA was extracted and mRNA was reverse transcribed to cDNA. Next, the VHH repertoire was isolated in 3 subsequent PCR reactions. First, primers CALL001 (5′-GTC CTG GCT GCT CTT CTA CA A GG-3′) and CALL002 (5′-GGT ACG TGC TGT TGA ACT GTT CC-3′) were used to isolate and separate heavy chain sequences that miss the CH1 exon (hcAb-specific) from CH1-bearing sequences (conventional heavy chains). Next, VHH genes were amplified with two subsequent nested PCRs with Forward primers SM017 and SM018 (5′-CCA GCC GGC CAT GGC TCA GGT GCA GCT GGT GGA GTC TGG-3′and 5′-CCA GCC GGC CAT GGC TGA TGT GCA GCT GGT GGA GTC TGG-3′, respectively) and Reverse primer CALL002 (1. Nested PCR), as well as Forward primer A4short (5′-CAT GCC ATG ACT CGC GGC CAC GCC GGC CAT GGC-3′) and Reverse Primer 38 (5′-GGA CTA GTG CGG CCG CTG GAG ACG GTG ACC TGG GT-3′; 2. Nested PCR). The VHH library was then subcloned into pHen4 phagemid vector to create surface-displayed VHH fusion proteins with the phage protein pIII. The phage library was then bio-panned for CA-specific binders in 3 subsequent panning rounds, followed by antigen recognition testing (phage-ELISA) of individual clones.

### Plasmids and Proteins

For bacterial expression of nanobodies, sequences were cloned into the pHen6 vector, thereby adding a C-terminal 6×His-tag for IMAC purification as described previously [31]. The expression plasmid for CA has been described previously [32], [33]. Additional CA variants (N-terminal CA domain [CA_NTD_; residues 1–146], C-terminal CA domain [CA_CTD_; 146–231]) were obtained by PCR cloning from pET11c-CA and confirmed by sequence analysis.

For protein production, *E. coli* BL21(DE3) CodonPlus-RIL (Stratagene) was used. Expression and purification CA_NTD_nb1 was carried out as described previously [31]. Full-length CA was purified as described [32], [33], [34]. For functional characterization in mammalian cells, translational fusions of CA_NTD_cb1 with mCherry and eGFP were constructed. The corresponding VHH genes were PCR-amplified with Primers

VHH F (5′-GGGGAGATCTCCATGGCGATCGCGCAGGTGCAGCTG-3′) and VHH R (5′-CCGGGCGGCCGCTGGAGACGGTGACCTGGGT-3′) containing AsiSI and NotI restriction sites, respectively, digested with AsiSI and NotI and ligated into pEGFP-N1 and a modified pEYFP-N1 vector (Clontech, CA, USA), where the YFP sequence had been replaced by the mCherry coding region. Intracellular Gag localization and interaction with CA_NTD_cb1 was determined using non-infectious HIV-1 plasmids either untagged (pcHIV) or as a 1∶1 co-transfection with a plasmid encoding eGFP embedded within the MA domain of *gag* (pcHIV.eGFP) [7], [8].

### QCM Affinity Measurements

Quartz Crystal Microbalance measurements were performed on an Attana A100 instrument. The ligand (purified CA_NTD_nb1) was covalently immobilized on a Carboxyl surface via NHS esterification according to the manufacturers' instructions. Purified full-length CA protein was run over the surface in decreasing concentrations (10, 5, 2.5, 1.25, 0.6125 µg/ml; triplicate injections). Contact time for association was set to 80 seconds. Dissociation was recorded for 220 seconds. Data fitting and rate constant calculation was carried out with ClampXP software.

### Cells

HeLa-Kyoto cells (a HeLa cell line characterized by little cell motility and thus high suitability for time lapse imaging) were maintained in DMEM supplemented with 10% fetal calf serum and gentamycin at 50 µg/ml (PAA, Germany). Transfection was performed using polyethylenimine (PEI; Sigma, Germany). To generate DNA/PEI complexes for transfection in 6-well plates, 2 µg DNA were mixed with 6 µg PEI in 150 µl DMEM per well, incubated for 15 minutes and added to the cells. For Confocal Microscopy 10^6^ cells were seeded on gridded 18×18 mm coverslips in a 6-well plate format. For cotransfection, 1∶1∶1 plasmid mixtures of pcHIV.eGFP/pcHIV and mCherry (negative control) or CA_NTD_cb1mCherry were prepared. For visualization of untagged HIV-1 Gag, a 1∶1 mixture of pcHIV and CA_NTD_cb1eGFP plasmid was prepared. 18 hours post transfection, cells were washed, fixed with 3,7% formaldehyde/PBS, permeabilized with 0,5% Triton X-100/PBS, DAPI (Invitrogen, USA) stained and mounted in Vectashield anti-fading reagent (Vector Laboratories, USA) on object slides. For 3D-SIM, HeLa-Kyoto cells were co-transfected with pcHIV and CA_NTD_cb1eGFP. 3D-SIM requires extensive laser excitation, easily causing fluorophore bleaching. For eGFP signal stabilization, cells were therefore stained with GFP-booster (ChromoTek, Germany) according to the manufacturer's protocol. HeLa-Kyoto cells, stably expressing CA_NTD_cb1eGFP were generated by stable transfection, followed by single cell sorting, clonal cultivation and tested by Western Blot analysis with a monoclonal α-GFP antibody (Roche, Switzerland). Stable cell lines were maintained in DMEM supplemented with 10% fetal calf serum, gentamycine at 50 µg/ml (PAA, Germany) and G418 at 1 mg/ml (PAA, Germany). For live cell analysis, 10^4^ cells were seeded in ibidi μ-slides (Ibidi, Germany). During acquisition, cells were maintained in phenol red-free, HEPES buffered DMEM (PAA, Germany).

### Confocal Microscopy

Colocalization analysis with fixed cells was performed with a confocal laser scanning microscope (TCS SP5/AOBS; Leica), using a UV-transmitting HCX PL 63×/1.4 oil objective. Fluorophores were excited with a 405 nm diode laser (4′,6-diamidino-2-phenylindole) 488-nm Ar laser line (eGFP) and a 561-nm diode pumped solid state laser line (mCherry). Images were recorded with a frame size of 512×512 pixels, a pixel size of 50 nm and the pinhole opened to 1 Airy unit.

### 3D-Structured Illumination Microscopy

3D-SIM was performed with an DeltaVision OMX v3 (Applied Precision) equipped with 405, 488 and 593 nm laser diodes, a 100×/1.4 NA Plan-Apochromat oil objective lens (Olympus) and Cascade II:512 EM CCD cameras (Photometrics). Samples were illuminated by directing coherence-scrambled laser light through a movable optical grating, generating a fine-striped illumination pattern on each plane. By moving the stage in z direction (125 nm steps), 15 images per z-section (five phases, three angles) were acquired and computationally processed to obtain a 3D dataset with a twofold enhanced optical resolution compared with conventional light microscopy [21], [22].

### Live Cell Confocal Microscopy

Live cell imaging of individual cells was performed on an UltraVIEW VoX spinning disc microscope (PerkinElmer) assembled to an Axio Observer D1 stand (Carl Zeiss, Inc.), equipped with a 63×/1.4 NA Plan-Apochromat oil immersion objective and a heated environmental chamber set to 37°C and CO2 perfusion set to 5%. Fluorophores were excited with the 488 nm laser line. Individual cells were recorded 8 hours post transfection for 1.5–2.5 h at 1 min time intervals and 12 z-sections per time point, covering 6 µm in z direction (500 nm steps). Z-stack projections were performed using Volocity software. For FRAP analysis, typically one third of the cell (covering membranous and cytoplasmic regions) was photobleached using two iterations of the Argon laser line set to 100% transmission. 24 prebleach frames (12 frames/min) and 84 postbleach frames (initial speed of 12 frames/min for the first 12 frames, followed by intervals of 6 frames/min) were recorded. Quantitative analysis was performed with ImageJ. Fluorescence recovery in membranous and cytoplasmic regions of interest was measured as the percentage of postbleach intensity compared to prebleach intensity. At least 7 cells were evaluated and the corresponding standard deviations were determined.

### Live Cell Widefield Microscopy and High-Content Analysis

Long-term visualization of HIV-1 Gag assembly in living cells was analyzed using an InCell Analyzer 1000 (GE Healthcare) equipped with a 20×/0.45 Plan-Fluor air objective. Images of living cells, seeded in Ibidi μ-slides, were automatically acquired from 12 different positions with exposure times of 500 ms for eGFP fusion proteins using standard filter HQ480/40. Quantitative image analysis was performed using INCell Developer Toolbox 1.7. Cells were segmented and individually identified by setting an intensity-based threshold. Next, the average number of granule-like structures per GFP positive cell was determined by size- and intensity-based threshold segmentation. Data were statistically evaluated, and visualized using Microsoft Excel.

## Supporting Information

Movie S1 **Visualizing the formation of HIV-1.** The time series corresponds to Figure 5. Shown is a HeLa-Kyoto cell, expressing CA_NTD_cb1eGFP and HIV-1 Gag, monitored for 90 min at 1 min time intervals. On the left, a projection of 12 z-sections is shown. On the right, a confocal mid section is shown.
(AVI)
